# Rheumatic manifestations of Chikungunya virus infection: Prevalence, patterns, and enthesitis

**DOI:** 10.1371/journal.pone.0249867

**Published:** 2021-04-22

**Authors:** Saovanee Benjamanukul, Manathip Osiri, Jira Chansaenroj, Chintana Chirathaworn, Yong Poovorawan

**Affiliations:** 1 Banphaeo General Hospital, Samutsakhon, Thailand; 2 Division of Rheumatology, Department of Medicine, Faculty of Medicine, Chulalongkorn University, Bangkok, Thailand; 3 Center of Excellence in Clinical Virology, Faculty of Medicine, Chulalongkorn University, Bangkok, Thailand; 4 Department of Microbiology, Faculty of Medicine, Chulalongkorn University, Bangkok, Thailand; 5 Tropical Medicine Cluster, Chulalongkorn University, Bangkok, Thailand; CEA, FRANCE

## Abstract

Chikungunya virus (CHIKV) is an arthropod-borne virus transmitted by mosquitoes of the genus *Aedes*. CHIKV infection causes various rheumatic symptoms, including enthesitis; however, these effects are rarely investigated. The aim of this study was to describe the rheumatic manifestations in CHIKV infection, estimate the prevalence of enthesitis in CHIKV-infected patients, and determine the factors associated with CHIKV-induced enthesitis. We conducted a prospective, observational study in patients with CHIKV infection confirmed by positive RT-PCR or IgM assay from October 2019 to March 2020. Patients with pre-existing inflammatory rheumatic diseases were excluded. A rheumatologist evaluated the demographic and clinical characteristics of the patients, including the number of inflamed joints, enthesitis sites, tendinitis, and tenosynovitis. The Leeds enthesitis index (LEI) and the Maastricht ankylosing spondylitis enthesis score (MASES) were used to evaluate enthesitis sites. Factors associated with enthesitis were determined using logistic regression analysis. One hundred and sixty-four participants diagnosed with CHIKV infection were enrolled. The mean (SD) age of the patients was 48.2 (14) years. The most common pattern of rheumatic manifestations was polyarthritis with or without enthesitis. Enthesitis was observed in 63 patients (38.4%). The most common site of enthesitis was the left lateral epicondyle as assessed by LEI and the posterior superior iliac spine as assessed by MASES. Multivariate analysis indicated that the number of actively inflamed joints and Thai-HAQ score at the initial evaluation were significantly associated with the presence of enthesitis. The main rheumatic manifestations of CHIKV infection were arthritis/arthralgia, with enthesitis as a prominent extraarticular feature. CHIKV infection can cause enthesitis at peripheral and axial sites. We found that enthesitis was associated with a high number of inflamed joints and reduced physical function. These results indicate that the assessment of enthesitis should be considered when monitoring disease activity and as a treatment response parameter in CHIKV-infected patients.

## Introduction

Chikungunya virus (CHIKV) is an arthropod-borne virus transmitted by mosquitoes of the genus *Aedes* (*A*. *albopictus and A*. *aegypti*) [1]. “Chikungunya” is a Makonde phrase meaning “that which bends up” and refers to the stooping posture displayed by CHIKV-infected patients as a result of excruciating joint pain [2]. The classic symptoms of CHIKV infection include an abrupt onset of fever, rash, and incapacitating joint pain. Clinically, CHIKV infection may lead to a broad spectrum of chronic rheumatic symptoms similar to rheumatoid arthritis (RA), spondyloarthritis (SpA), undifferentiated arthritis (UA), psoriatic arthritis, and soft-tissue rheumatism [3–5]. In individual patients, arthralgia or arthritis may persist for months or years. Persistent symptoms can significantly impair the quality of life of patients [6]. In addition to arthritis, the reported rheumatic manifestations for CHIKV infection include tendinitis, tenosynovitis, neuropathic pain, and, rarely, enthesitis.

The first CHIKV outbreak in Thailand was reported in 1958 [7]. The second was in 2008–2009 and affected mainly the southern region of the country [8, 9]. However, in 2018, a large-scale outbreak occurred in different parts of Thailand. Although the clinical manifestations and cytokine profiles of CHIKV infection have been reported [10–13], no study has investigated the rheumatic manifestations associated with infection by this virus. The objectives of our study were to describe the rheumatic manifestations of CHIKV infection, estimate the prevalence of enthesitis in CHIKV-infected patients, and determine the factors associated with the presence of CHIKV infection-induced enthesitis.

## Materials and methods

This study was conducted according to the principles expressed in the Declaration of Helsinki and Good Clinical Practice (ICH-GCP) guidelines. The proposal was approved by the Institutional Review Board of the Faculty of Medicine, Chulalongkorn University (IRB No. 798/62). All the study participants were informed of the study objectives and provided written consent.

### Study area and populations

Banphaeo is a district of Samutsakhon province, located approximately 70 km southwest of Bangkok, Thailand. The average population density is 410 people per square kilometer, and most are agriculturists. The 2019–2020 CHIKV outbreak began at the end of the rainy season in 2019 (October) and lasted until the end of the winter in 2020 (March). At the onset of illness, adult patients suspected of being infected with CHIKV were routinely assessed by general practitioners. These patients were later referred to a rheumatologist (SB) for the evaluation of rheumatologic conditions and further management. Adult patients (≥18) with rheumatic symptoms and with CHIKV infection confirmed by positive real-time RT-PCR or IgM results were included in this study. Real-time RT-PCR and IgM testing for CHIKV diagnosis have been described elsewhere [14]. Patients who were unable to give consent or those with pre-existing inflammatory rheumatic diseases were excluded.

### Data collection and clinical evaluation

In this observational prospective cohort study, we collected disease symptom data (fever, rash onset, pain score, and joint pain locations) of the patients at their first visit to a general practitioner. All the participants were followed up by a rheumatologist (SB) every one-to-two months, depending on the severity of the pain or arthritic symptoms. They were assessed for their personal history, body mass index (BMI), underlying diseases, pre-existing joint diseases, pain score, tender and/or swollen joint count (66/68), and presence and sites of tendinitis, enthesitis, dactylitis, and tenosynovitis. The pain score was assessed using a verbal numeric scale in which patients were asked to verbally state a number between 0 (no pain) and 10 (worst possible pain) that corresponded to their present pain intensity. Enthesitis was defined as the presence of at least one tender entheseal site using the Leeds enthesitis index (LEI) and the Maastricht ankylosing spondylitis enthesis score (MASES). Neuropathic pain was assessed using the Thai version of the Douleur Neuropathique 4 questions (DN4) questionnaire [15], and the presence of neuropathic pain was defined as a score ≥4. Other rheumatic symptom data collected included back pain and myalgia. At the first visit, the rheumatologist performed a functional evaluation using the Thai version of the Health Assessment Questionnaire (Thai-HAQ) [16] and a quality-of-life evaluation using the Thai version of the standardized EQ-5D-5L questionnaire [17] at the onset of symptoms. The acute phase of CHIKV infection was defined as having symptoms for less than or equal to three weeks; the post-acute phase as having symptoms for between three weeks and three months; and the chronic phase as having symptoms for more than three months [18]. Patients who progressed to the chronic stage were classified as having post-CHIKV chronic inflammatory rheumatism (pCHIK-CIR) and post-CHIKV musculoskeletal disorder (pCHIK-MSD) [3]. pCHIK-CIR was classified into the following three categories: 1) RA, based on the 2010 American College of Rheumatology/European League Against Rheumatism (ACR/EULAR) classification criteria [19]; 2) SpA, as defined by the European Spondyloarthropathy Study Group (ESSG) [20]; and 3) UA, when none of the existing classification criteria for definitive diagnoses were fulfilled [21]. pCHIK-MSD was categorized as locoregional or diffuse (i.e., at least 4 painful areas) [3]. Data regarding the medication prescribed at the acute, post-acute, and chronic phases were also collected.

### Statistical analysis

Categorical variables were summarized using percentages. Continuous variables were shown as means ± standard deviation (SD) and/or medians with interquartile range (IQR) as appropriate. The demographic and clinical parameters associated with enthesitis were analyzed using logistic regression. Associations between LEI and MASES scores were expressed using Spearman’s rank correlation coefficient. Data were analyzed using Stata statistical software version 15.1 (StataCorp, College Station, TX, USA).

## Results

A total of 209 patients were assessed by general practitioners as fulfilling the diagnosis criteria for acute CHIKV infection. Forty-two patients were lost to follow-up before assessment by a rheumatologist. Three patients were excluded because of pre-existing inflammatory rheumatic disorders, i.e., RA in 2 cases and SpA in 1. Consequently, this study reported the data for 164 patients.

CHIKV diagnosis was confirmed by RT-PCR in 92 patients (56%) and by IgM antibody assay in 72 patients (44%). The demographic and clinical data of the enrolled patients are presented in Table 1. The mean (SD) age of the patients was 48.2 (14) years, with a female to male patient ratio of 2.8:1. Sixty-eight patients (41.4%) had comorbid diseases, including hypertension in 35 patients (21.3%), diabetes mellitus in 16 patients (9.8%), and asthma in 11 patients (6.7%). Sixteen patients (9.8%) were hospitalized for severe pain. No deaths were reported in our study. The median (IQR) duration of work absenteeism was 7 days (4–10).

**Table 1:** The baseline characteristics of participants with rheumatic manifestations resulting from Chikungunya virus infection at the rheumatologist’s first assessment (n = 164).

Characteristics	Mean ± SD
Age (years)	48.2 ± 14 (19–90)
Female, no. (%)	121 (73.8)
Body mass index (kg/m^2^)	26 ± 5.7
Active smokers, no. (%)	8 (4.9)
Actively inflamed joint count (tender and/or swollen)	7.8 ± 8.9 (median 4) (IQR 1–12)
Presence of enthesitis, no. (%)	63 (38.4)
LEI	1.6 ± 1.4 (median 1) (IQR 1–2)
MASES	1.8 ± 2.2 (median 2) (IQR 0–3)
Presence of tendinitis, no. (%)	54 (32.9)
Presence of tenosynovitis, no. (%)	5 (3)
Presence of back pain, no. (%)	72 (43.9)
Neuropathic pain, no. (%)	56 (34.1)
Thai-HAQ score	2.01 ± 0.84
EQ-5D-5L score	0.27 ± 0.32 (median 0.23) (IQR 0.01–0.54)
Comorbidities, no. (%)	68 (41.4)

LEI = Leeds enthesitis index; MASES = Maastricht ankylosing spondylitis enthesis score; Thai-HAQ = Thai version of the Health Assessment Questionnaire; EQ-5D-5L = EuroQoL-5 Dimension-5 Level.

Data are presented as means ± SD for continuous outcomes.

The information of the clinical features at the onset of CHIK infection as assessed by general practitioners showed that rashes were reported in 136 patients (82.9%), and the mean (SD) duration of fever before the occurrence of rash was 2 (2.6) days (median [IQR] 1 [0–3]). Rheumatic manifestations occurred within two weeks of fever onset with a mean (SD) of 0 (2.6) days (median [IQR] 0 [0–0]). Based on the general practitioners’ examinations, the most common pattern of articular involvement was a polyarticular form in 152 patients (92.7%), followed by an oligoarticular form in 10 patients (6.1%) and a monoarticular form in 2 (1.2%). Joint pain affected both the upper and lower extremities in 129 patients (79%). Symmetrical joint pain was observed in 155 patients (94.5%), and both small and large joints were involved in 150 patients (91.5%). The most common site of joint pain was the ankle (*n* = 133, 81.1%), the proximal interphalangeal (PIP) joint of the finger (*n* = 125, 76.2%), the wrist (*n* = 118, 72%), and the knee (*n* = 112, 68.3%). The mean (SD) pain score was 8.1 (1.8). The mean (SD) number of actively inflamed joints (tender and/or swollen) at the acute stage was 21.0 (14.1). Nonsteroidal anti-inflammatory drugs (NSAIDs) were prescribed for 51 patients (31.1%); combination treatment with NSAIDs and/or muscle relaxant and/or tramadol and/or paracetamol was prescribed for 48 patients (29%). Paracetamol alone was prescribed for 30 patients (18.3%), muscle relaxant alone for 9 patients (5.5%), prednisolone alone for 3 patients (1.8%), and tramadol alone for 12 patients (1.3%). The medication prescribed for 8 patients (4.9%) was unknown because they received treatment from private clinics. Three patients were not prescribed any medication.

The median (IQR) duration from a general practitioner’s visit to a rheumatologist consultation was 21 days (12–39) (with a mean [SD] of 28.6 [23] days). Eighty-three patients (50.6%) were in the acute phase when they were assessed by a rheumatologist, 78 (47.6%) in the post-acute phase, and 3 (1.8%) in the chronic phase. The patterns of joint and/or enthesis involvement at the rheumatologist’s first assessment are shown in Table 2. Isolated joint involvement was the most common form of rheumatic manifestation with CHIKV infection, affecting 86 patients (52.4%). Polyarthritis was predominant in 36 patients (22%). Fifty-seven patients (34.8%) displayed both joint and enthesis involvement, while 16 patients (9.8%) presented with polyarthritis and enthesitis in at least three sites. The median (IQR) number of actively inflamed joints (tender and/or swollen joint count) examined by the rheumatologist at the first appointment was 4 (1–12) (mean ± SD: 7.8 ± 8.9 joints). The median (IQR) tender joint count was 4 (1–10) (mean ± SD: 7.1 ± 9 joints), while the median (IQR) swollen joint count was 2 (0–6) (mean ± SD: 4.1 ± 5.7 joints). The most frequently affected joint examined by the rheumatologist was the PIP joint of the finger (*n* = 89, 54.3%), followed by the ankle (*n* = 72, 43.9%), wrist (*n* = 68, 41.5%), metacarpophalangeal (MCP) joint (*n* = 51, 31.1%), and knee (*n* = 27, 16.5%). Uncommon sites of joint involvement included the acromioclavicular joint in 22 patients (13.4%), sternoclavicular joint in 9 patients (5.5%), and temporomandibular joint in 5 patients (3%).

**Table 2:** Patterns of joint and/or entheseal involvement at the first assessment by a rheumatologist (n = 164).

Characteristic	Number (%)
**Joint involvement only**	**86 (52.4)**
Monoarthralgia/Monoarthritis	9 (5.5)/6 (3.7)
Oligoarthralgia/Oligooarthritis	9 (5.5)/14 (8.5)
Polyarthralgia/Polyarthritis	12 (7.3)/36 (22)
**Joint and entheseal involvement**	**57 (34.8)**
In less than or equal to two sites	30 (18.3)
Monoarthralgia/Monoarthritis	1 (0.6)/3 (1.8)
Oligoarthralgia/Oligooarthritis	4 (2.4)/3 (1.8)
Polyarthralgia/Polyarthritis	5 (3)/14 (8.5)
In at least three sites	27 (16.5)
Monoarthralgia/Monoarthritis	1 (0.6)/0
Oligoarthralgia/Oligooarthritis	2 (1.2)/1 (0.6)
Polyarthralgia/Polyarthritis	7 (4.3)/16 (9.8)
**Entheseal involvement only**	**6 (3.7)**
In less than or equal to two sites	4 (2.4)
In at least three sites	2 (1.2)
**Neuropathic pain, resolved arthritic symptoms**	**4 (2.4)**
**Complete recovery**	**11 (6.7)**

At the first assessment by a rheumatologist, joint pain due to acute CHIKV infection had intrinsic joint and/or tendon and/or tenosynovial and/or enthesis involvement in 97 patients (59%). Joint involvement only was reported in 62 patients (38%), while tendinitis and/or tenosynovitis and/or enthesitis only was reported in 5 patients (3%). Tendinitis was detected in 54 patients (32.9%) and tenosynovitis in 5 (3%). Achilles tendinitis was predominant in patients with tendinitis (48 patients; 88.9%).

**Fig 1:**
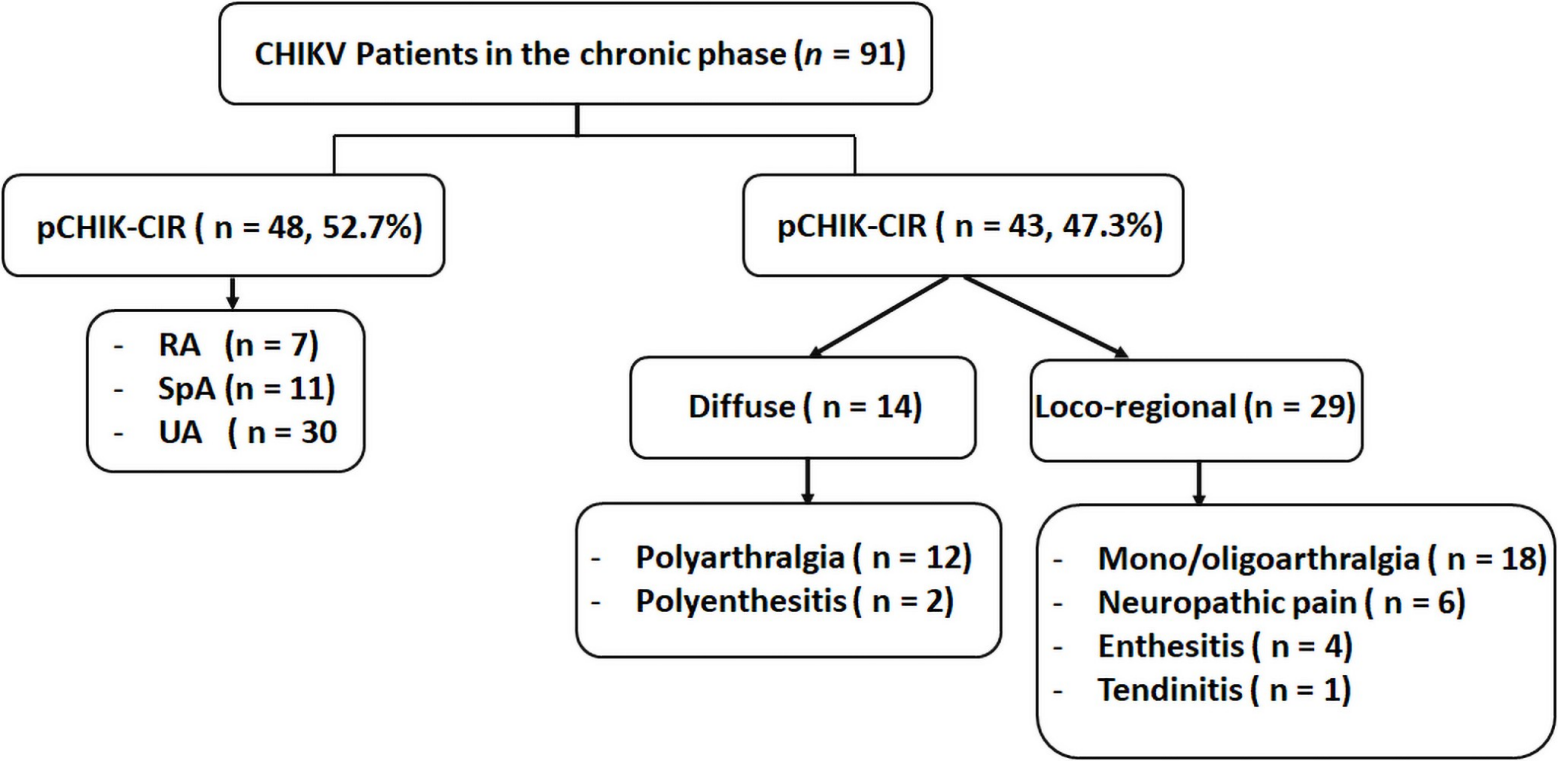
Rheumatic manifestations in patients with chronic CHIKV infection. CHIKV, Chikungunya virus; pCHIK-CIR, post-chikungunya chronic inflammatory rheumatism; pCHIK-MSD, post-chikungunya musculoskeletal disorder; RA, rheumatoid arthritis; SpA, spondyloarthritis; UA, undifferentiated arthritis.

The prevalence of chronic CHIKV infection in our cohort was 55.5% (91 out of 164 patients). The rheumatologic manifestations in these patients are shown in Fig 1. Forty-eight patients (52.7%) had pCHIK-CIR, including 7 (14.6%), 11 (22.9%), and 30 (62.5%) who were classified as having RA, SpA, and UA, respectively. Forty-three patients (47.3%) had pCHIK-MSD. Of these, 14 (32.6%) displayed diffuse symptoms (polyarthralgia in 12 patients and polyenthesitis in 2). Twenty-nine (67.4%) patients had locoregional symptoms (monoarthralgia/oligoarthralgia in 18 patients, neuropathic pain in 6, enthesitis in 4, and tendinitis in 1).

Enthesitis was observed in 63 patients (38.4%). Thirty-seven (58.7%), 25 (39.7%), and 1 (1.6%) patients presented with enthesitis in the acute, post-acute, and chronic phases, respectively, at the first assessment by a rheumatologist. LEI values and MASESs are presented in Tables 3 and 4. The mean (SD) LEI value was 1.6 (1.4). The most common enthesitis site as assessed by the LEI was the left lateral epicondyle (42.9%), and the left medial femoral condyle (38.1%) was the second. The mean (SD) MASES was 1.8 (2.2). The most common sites determined using the MASES were the posterior superior iliac spine (PSIS; 38.1% of patients) and fifth lumbar spinous process (17.5% of patients). The LEI was shown to be poorly associated with the MASES (*r* = 0.14).

**Table 3:** The distribution of entheseal sites as assessed using the Leeds enthesitis index (LEI) at the first assessment by a rheumatologist (n = 63).

Entheseal site	*N* (%)
Lateral epicondyle, right	20 (31.7)
Lateral epicondyle, left	27 (42.9)
Medial femoral condyle, right	20 (31.7)
Medial femoral condyle, left	24 (38.1)
Achilles tendon insertion, right	5 (7.9)
Achilles tendon insertion, left	6 (9.5)

**Table 4:** The distribution of entheseal sites as assessed using the Maastricht Ankylosing Spondylitis Enthesis Score (MASES) at the first assessment by a rheumatologist (n = 63).

Entheseal site	*N* (%)
First costochondral joint, right	5 (7.9)
First costochondral joints, left	5 (7.9)
Seventh costochondral joint, right	5 (7.9)
Seventh costochondral joint, left	5 (7.9)
Posterior superior iliac spine (PSIS), right	24 (38.1)
Posterior superior iliac spine (PSIS), left	24 (38.1)
Anterior superior iliac spine (ASIS), right	6 (9.5)
Anterior superior iliac spine (ASIS), left	6 (9.5)
Iliac crest, right	9 (14.3)
Iliac crest, left	9 (14.3)
Proximal insertion of the Achilles tendon, right	5 (7.9)
Proximal insertion of the Achilles tendon, left	6 (9.5)
Fifth lumbar spinous process	11 (17.5)

Back pain was detected in 72 patients (43.9%) and myalgia in 118 (72%). Diffuse myalgia was the major complaint for 70% of patients, and local myalgia in the calf muscle was found in 3%. Fifty-six patients (34.1%) in all phases reported having neuropathic pain as assessed by the DN4 score. The characteristics of neuropathic pain included tingling in 52.9% of patients, pins and needles in 50.6%, electric shock in 31%, itching in 31%, burning in 27.7%, numbness in 18.7%, hypoesthesia to touch in 23.3%, hypoesthesia to pinprick in 18.7%, and increased pain by brushing in 5.8%. Neuropathic pain in the hands was found in 85.7% of patients, 25% reported neuropathic pain in the feet, and 17.9% reported neuropathic pain in both hands and feet. Neuropathic pain displayed a glove- and/or stocking-like pattern in 96.4% of our patients, and presented as carpal tunnel syndrome in 3.6%. Two-thirds (32/48) of patients with neuropathic pain reported a greater than 50% improvement after taking 300 mg of gabapentin per day. Physical function and quality of life at illness onset were moderately impaired, with a mean (SD) Thai-HAQ score of 2.01 (0.84) and a mean (SD) value for the Thai version of the EQ-5D-5L utility of 0.27 (0.32) (median [IQR] 0.23 [0.01–0.54]).

The treatments administered to patients in the post-acute and chronic phases are shown in Table 5. The treatments for the post-acute phase of CHIKV infection with which the patients showed a greater than 50% improvement in pain score at one-month follow-up included NSAIDs only (90%; 44/49), low-dose (10 mg or less per day) prednisolone (84.4%; 27/32), chloroquine (71.1%; 27/38), and conventional synthetic Disease-Modifying Anti-Rheumatic Drugs (csDMARDs; 50% [2/4]). Patients who received chloroquine, prednisolone, or DMARDs were also prescribed NSAIDs for pain relief. The treatment response assessment for the chronic phase after three months of infection has yet to be determined.

**Table 5:** Treatment for rheumatic involvement in patients in the post-acute and chronic phases of Chikungunya infection.

Treatment	Post-acute phase (*n* = 138) *	Chronic phase (*n* = 91) **
1 DMARD	-	2 SSZ
2 DMARDs	3 MTX + CQ, 1 SSZ + CQ	6 MTX + CQ, 3 SSZ + CQ
3 DMARDs	-	8 MTX + SSZ + CQ
		3 MTX + CQ + prednisolone
Chloroquine only	38	30
NSAIDs only	49	26
Oral prednisolone only (≤10 mg/day)	32	3
Chloroquine and prednisolone (≤10 mg/day)	6	4
Tramadol	1	-
Paracetamol	2	-
Gabapentin only	6	6

Abbreviations: DMARDs = disease-modifying antirheumatic drugs; MTX = methotrexate; SSZ = sulfasalazine; CQ = chloroquine; NSAIDs = nonsteroidal anti-inflammatory drugs.

* From the first visit to a rheumatologist in 78 patients; evolved from the acute phase in 64 patients; complete recovery before rheumatologist assessment in 4 patients.

** From the first visit to a rheumatologist in 3 patients; evolved from the acute phase to the post-acute phase to the chronic phase in 35 patients; evolved from the post-acute phase to the chronic phase in 53 patients.

Table 6 shows the univariate and multivariate logistic regression analysis for factors associated with the presence of enthesitis. In the univariate analysis, three factors were significantly associated with enthesitis, including neuropathic pain, the number of actively inflamed joints as assessed by a rheumatologist, and the Thai-HAQ score at disease onset. However, in the multivariate analysis, only the number of actively inflamed joints (odds ratio [OR] 1.11, 95% CI 1.05–1.16, *p*<0.001) and the Thai-HAQ score at illness onset (OR 1.71, 95% CI 1.06–2.76, *p* = 0.03) were significantly associated with the presence of enthesitis.

**Table 6:** Factors associated with the presence of enthesitis.

Parameter	Univariate associations		Multivariate associations	
	OR (95% CI)	*p*-value	OR (95% CI)	*p*-value
Age	1.01 (0.98–1.02)	0.71		
Gender	1.22 (0.59–2.53)	0.58		
BMI	1.03 (0.98–1.09)	0.23		
Neuropathic pain	2.08 (1.07–4.03)	0.03		
The number of actively inflamed joints	1.11 (1.06–1.17)	<0.001	1.11 (1.05–1.16)	<0.001
Thai-HAQ score	2.04 (1.33–3.14)	0.001	1.71 (1.06–2.76)	0.03

OR = odds ratio; CI = confidence interval; BMI = body mass index; Thai-HAQ = Thai version of the Health Assessment Questionnaire.

## Discussion

This is the first study to report the rheumatic manifestations associated with confirmed cases of CHIKV infection in Thailand, including detailed data on enthesitis.

In the acute phase, joint involvement was mostly symmetric, involving both small and large joints of the upper and lower limbs. Polyarthritis was the most common manifestation of articular involvement, which was in agreement with that reported in several studies [22–25], but different from the results obtained in another study [26], in which oligoarticular involvement was predominant. The affected joints examined by general practitioners at the acute stage were different from those assessed by the rheumatologists, which might be explained by the different assessment times or differences in the ability to discriminate between articular and periarticular involvement.

In the acute phase of the disease, CHIKV replicates in the target tissues, especially the synovium, periosteum, and skin, all of which contain fibroblasts [2, 27]. Innate and adaptive immune responses contribute to the occurrence of CHIKV infection-induced arthritis [1]. Monocytes and macrophages play a significant role in the immunopathology of CHIKV-related arthritis, and both are important for efferocytosis and the resolution of inflammation [28]. Several cytokines and chemokines induced by inflammation are associated with the severity and/or persistence of CHIKV infection-related symptoms [29]. The cytokine profile in CHIKV infection is similar to that in inflammatory arthritis [1, 30, 31], which may explain the similarities between the pathogenesis and clinical features of CHIKV-associated arthropathy and those of RA and SpA. Proinflammatory cytokines such as tumor necrosis factor (TNF), interleukin 1 beta (IL-1β), IL-6, and IL-17 also mediate pain by directly acting on the nociceptive system [32]. TNF and IL-6 can also function as mediators of neuropathic pain [33–35].

The prevalence of chronic CHIKV infection varies between 5.9% and 84.3%, with the time at the assessment ranging from 3 to 81 months [5, 25, 36–44]. This variation depends on the definition of chronic CHIKV, the assessor’s specialty, and the assessment time. The prevalence of chronic CHIKV in our cohort was 55.5% at three-month evaluation, which was higher than that reported in previous studies with a similar time of assessment (27.5%–40.2%) [5, 45].

Few studies have reported on enthesitis in CHIKV infection [46]. Enthesis is defined as the area where a tendon, ligament, or joint capsule inserts into the bone and acts to transmit the tensile load from soft tissues to the bone. The tissue in this transitional zone is composed of fibrocartilage, and is populated by fibroblasts [47]. The prevalence of enthesitis in our study was 38.4%, which was higher than that reported in other studies [3, 5]. A prevalence of 2.7% was reported for the acute to subacute phases [5] and between10.1% and 19.4% for the chronic phase. However, these studies did not provide detail on the anatomical sites of enthesitis [3, 5], with only one case report having described the presence of enthesitis at the insertions of both Achilles tendons in CHIKV infection [46]. Additionally, entheseal sites in CHIKV infection, as assessed by LEI or MASES, have never been reported. The LEI, which was developed specifically for psoriatic arthritis, is simple to use as only six sites are assessed. However, because it lacks discriminative power as only peripheral entheseal sites are evaluated, we also used the MASES, which is suitable for use with ankylosing spondylitis (AS) and axial SpA [48, 49]. As assessed by MASES, the most frequent entheseal site in our study was the PSIS, which was different from that assessed in a study on psoriatic arthritis, where the most commonly reported site was Achilles insertion [50]. LEI values showed only a very weak association with MASES in our research, suggesting that CHIKV infection involved both peripheral and axial entheseal sites. Both the LEI and MASES should be used for evaluating entheseal sites in CHIKV-infected patients. Other entheseal score indices may be further validated in CHIKV-infected patients.

The involvement of acromioclavicular, sternoclavicular, and temporomandibular joints was also observed in our study, as well as in a previous report [37, 51]. These joints are also involved early in AS [52] and psoriatic arthritis [50]. These synovial joints are lined with fibrocartilage rather than hyaline cartilage [53], and entheseal compartments are dominant within these joints [47].

Studies have reported that approximately 15%–23% of CHIKV-infected patients present with neuropathic pain [5, 26, 54]. In contrast, the prevalence of neuropathic pain in our patients was higher, at 34.1%. Peripheral axonal polyneuropathy was confirmed by nerve conduction studies in one report [55]. In another study, meanwhile, ultrasound examination indicated that only 14% of patients with hand paresthesia showed thickened median nerves [56]. Other mechanisms might explain neuropathic pain in CHIKV-infected patients, such as small nerve fiber involvement. Neuropathic pain in our patients displayed a glove- and/or stocking-like pattern, which was consistent with peripheral neuropathy rather than nerve entrapment syndrome. However, we did not perform nerve conduction velocity (NCV) tests or skin biopsies in our patients, and the mechanisms underlying the neuropathic pain were therefore not elucidated. Nevertheless, more than half of our patients who reported neuropathic pain showed an improvement of at least 50% with 300 mg per day of gabapentin treatment within one month of follow-up. Neuropathic pain in CHIKV-infected patients may be managed successfully with medications such as gabapentin, opioids (such as tramadol), or tricyclic antidepressants. Physicians should be aware if joint pain in CHIKV-infected patients does not respond well to the usual analgesics as the pain may be either nociceptive or neuropathic.

Pain improvement in the post-acute phase varied considerably with each treatment option. However, we could not compare the effectiveness of the different treatments owing to different baseline severity and the different types of rheumatic manifestations. A few studies have reported the medications prescribed in the post-acute phase of CHIKV infection, including NSAIDs, chloroquine, prednisolone, and DMARDs [57–59]. Ninety percent of our patients in the post-acute phase showed a good response to NSAIDs, which was consistent with the results of a previous study [57]. We recommend NSAIDs as the first-line treatment for CHIKV-infected patients without contraindications. Approximately 80% of our patients who were prescribed low-dose prednisolone experienced an improvement in pain score of at least 50% after one-month follow-up. We recommend using low-dose prednisolone with slow tapering for patients who do not respond well to NSAIDs or those who have a high arthritis or enthesitis severity score. French and Brazilian guidelines recommend that low-dose prednisolone, with slow and gradual reduction, can be used for intractable synovitis and tenosynovitis [18, 60]. Among the patients who were prescribed chloroquine, three-quarters exhibited an improvement in the pain score of at least 50% after one-month follow-up. It was not clear whether or not the rapid response to chloroquine in our patients was primarily due to the NSAIDs prescribed alongside chloroquine. The evidence for chloroquine efficacy in relieving pain in the post-acute phase was also inconclusive [59]. The Brazilian guidelines recommend using hydroxychloroquine for joint pain in the subacute phase [60]. These observations highlight that the efficacy of chloroquine requires further assessment. For the effectiveness of DMARDs in CHIKV-associated arthritis, half of our patients responded to DMARDs in the post-acute phase. These results corroborated those reported in a previous study, in which early methotrexate administration was not effective in improving outcomes in patients with post-CHIKV-infection arthritis [58]. However, a one-month follow-up is not long enough to evaluate the responses to DMARDs.

We found that approximately 83% of our patients developed skin rashes in the acute phase of the disease, which was higher than that reported in previous studies [26, 42, 61]. A high percentage of patients (82.9%) presenting with rashes were also diagnosed with painkiller allergies. To avoid misdiagnosis, physicians should be cautious when prescribing medications for patients with suspicion of CHIKV infection as the rashes could be either a manifestation of CHIKV infection or a drug allergy.

In terms of physical function and quality of life, the Thai-HAQ scores at the initial evaluation indicated moderate functional impairment, consistent with that previously reported [37]. The EQ-5D-5L index scores at the onset of symptoms were low, indicating that CHIKV infection impaired the quality of life of the patients. As the patients completed both questionnaires at the first visit to a rheumatologist and not when visiting a general practitioner, the Thai-HAQ and EQ-5D-5L scores at the onset of symptoms might be affected by recall bias.

Multivariate logistic regression analysis showed that the number of actively inflamed joints assessed by a rheumatologist and the Thai-HAQ score at the onset of symptoms were significantly associated with enthesitis. Highly active CHIKV-related disease was associated with enthesitis, similar to that found in psoriatic arthritis [62]. Enthesitis might be a sign of increased burden as it is associated with disease severity and poor function, which is consistent with that seen in SpA patients who also have enthesitis [63]. One study reported that the presence of at least one site of enthesitis increased the risk of developing chronic CHIKV-related arthritis [5]. To confirm this finding, enthesitis found in our patients should be followed up in the longer term, i.e., from three months onwards.

This study had some limitations. First, selection bias might have occurred because we only included CHIKV-infected patients seen at our hospital, and patients with mild, self-limited disease might not seek treatment or the disease may resolve before assessment by a rheumatologist. Therefore, our patients might not be representative of the entire CHIKV-infected population. Secondly, enthesitis was evaluated by clinical examination only, and its prevalence might have been underestimated owing to a lack of imaging confirmation.

In conclusion, rheumatic manifestations resulting from CHIKV infection comprise a broad spectrum of articular features mimicking several forms of chronic inflammatory arthritis, as well as enthesitis. Enthesitis usually occurs together with polyarthritis and is associated with increased arthritic severity and poorer physical function. Thus, enthesitis should be evaluated and followed up in all CHIKV-infected patients.
